# BioMaS: a modular pipeline for Bioinformatic analysis of Metagenomic AmpliconS

**DOI:** 10.1186/s12859-015-0595-z

**Published:** 2015-07-01

**Authors:** Bruno Fosso, Monica Santamaria, Marinella Marzano, Daniel Alonso-Alemany, Gabriel Valiente, Giacinto Donvito, Alfonso Monaco, Pasquale Notarangelo, Graziano Pesole

**Affiliations:** 1 Institute of Biomembranes and Bioenergetics, Consiglio Nazionale delle Ricerche, via Amendola 165/A, Bari, 70126 Italy; 2 Algorithms, Bioinformatics, Complexity and Formal Methods Research Group, Technical University of Catalonia, E-08034, Barcelona, Spain; 3 National Institute of Nuclear Physics, via E. Orabona 4, Bari, 70125 Italy; 4 Department of Biosciences, Biotechnology and Biopharmaceutics, University of Bari “A. Moro”, via E. Orabona 4, Bari, 70125 Italy; 5 Center of Excellence in Comparative Genomics, University of Bari “A. Moro”, via E. Orabona, 4, Bari, 70125 Italy

**Keywords:** Metagenomics, Bioinformatics, Microbiome, Meta-barcoding, High-Throughput Sequencing

## Abstract

**Background:**

Substantial advances in microbiology, molecular evolution and biodiversity have been carried out in recent years thanks to Metagenomics, which allows to unveil the composition and functions of mixed microbial communities in any environmental niche. If the investigation is aimed only at the microbiome taxonomic structure, a target-based metagenomic approach, here also referred as Meta-barcoding, is generally applied. This approach commonly involves the selective amplification of a species-specific genetic marker (DNA meta-barcode) in the whole taxonomic range of interest and the exploration of its taxon-related variants through High-Throughput Sequencing (HTS) technologies. The accessibility to proper computational systems for the large-scale bioinformatic analysis of HTS data represents, currently, one of the major challenges in advanced Meta-barcoding projects.

**Results:**

BioMaS (Bioinformatic analysis of Metagenomic AmpliconS) is a new bioinformatic pipeline designed to support biomolecular researchers involved in taxonomic studies of environmental microbial communities by a completely automated workflow, comprehensive of all the fundamental steps, from raw sequence data upload and cleaning to final taxonomic identification, that are absolutely required in an appropriately designed Meta-barcoding HTS-based experiment. In its current version, BioMaS allows the analysis of both bacterial and fungal environments starting directly from the raw sequencing data from either Roche 454 or Illumina HTS platforms, following two alternative paths, respectively. BioMaS is implemented into a public web service available at https://recasgateway.ba.infn.it/ and is also available in Galaxy at http://galaxy.cloud.ba.infn.it:8080 (only for Illumina data).

**Conclusion:**

BioMaS is a friendly pipeline for Meta-barcoding HTS data analysis specifically designed for users without particular computing skills. A comparative benchmark, carried out by using a simulated dataset suitably designed to broadly represent the currently known bacterial and fungal world, showed that BioMaS outperforms QIIME and MOTHUR in terms of extent and accuracy of deep taxonomic sequence assignments.

**Electronic supplementary material:**

The online version of this article (doi:10.1186/s12859-015-0595-z) contains supplementary material, which is available to authorized users.

## Background

Substantial advances in microbiology, molecular evolution and biodiversity have been reached in recent years thanks to Metagenomics, which allows an unprecedented large scale investigation of the composition and functions of mixed microbial communities in any environmental niche, plant or animal host, without the prerequisite to isolate or culture the single species. The composition of resident microbial species and their genetic capabilities can be both addressed by a shotgun HTS approach. However, if the purpose of the analysis is limited to investigate the taxonomic composition, an amplicon-based approach, through the PCR-targeted sequencing of selected genomic markers, is often more sensitive in species resolution and identification and less expensive in terms of both sequencing and computational analysis. The genetic markers used for taxonomic classification are commonly named “barcodes” and the metagenomic surveys based on them is here referred as Meta-barcoding. An ideal barcode should be ubiquitous in the taxonomic range of interest (e.g. Bacteria, Fungi, Metazoa) and include highly hypervariable regions, suitable for discriminating at lower taxonomic ranks (e.g. genus, species), flanked by highly conserved ones on which to focus the design of universal primers pairs able to work in a wide range of species, hopefully in the entire Kingdom of interest. Finally, its length must be consistent with that of the reads produced by the most recent versions of HTS platforms. The internal transcribed spacers 1 and 2 (ITS1 and ITS2) of the ribosomal RNA gene cluster and one or few hyper-variable regions of 16S ribosomal RNA gene are generally used to identify fungal and bacterial taxa, respectively. They are typically amplified by means of well-known taxonomically universal primers [1] with the resulting libraries processed through HTS technologies [1,2]. Thanks to the enormous improvement of the latter, Metagenomics is currently experiencing an unprecedented expansion of its applications and perspectives. Unfortunately, such biotechnological progress has not yet been adequately complemented by a comparable development of bioinformatics resources for handling and processing the large number (up to 10^9^) of 100–700 bp long sequences produced per run by HTS platforms. Indeed, even if the intrinsic error rate of HTS technologies, the read length and the throughput/coverage ratio surely affect the sensitivity of both taxon and gene annotation of metagenomic data, the most serious bottleneck is the availability of accurate and effective systems that allow a friendly and comprehensive large-scale bioinformatic assessment of produced reads. Undoubtedly, researchers involved in advanced metagenomics projects need both powerful computational infrastructures and, in most cases, robust informatics know-how in order to use and combine the most suitable tools needed for filtering, denoising, clustering, and assigning to taxonomic ranks the huge amount of sequence reads generated by HTS. These computational operations are all essential in order to obtain a consistent taxonomic classification starting from raw Meta-barcoding HTS data. Unfortunately, in common practice some of these steps are neglected resulting in the production of partial or incorrect inferences. A very common difficulty, often hard to overcome, is the integration of different analysis tools in a comprehensive workflow. Trivially, the conversion of the output of a bioinformatic analysis step in the right format to be subjected to the next one could be a tricky subject. Moreover, if huge amount of HTS data must flow through the entire process, as always happens in metagenomic projects, the computational power represents a remarkable limiting factor. As the basic strategy currently adopted to infer the taxonomic class of barcode sequences includes their comparison with already annotated sequences by means of similarity, composition or phylogeny based methods [3], another very critical issue is the absolute requirement of rich and properly annotated reference resources [4]. Finally, the possible occurrence of sequencing errors, specific for each HTS platform used in the experiment requires suitable preliminary steps for quality check and denoising in order to avoid misleading inferences.

BioMaS (*Bio*informatic analysis of *M*etagenomic *a*mplicon*S*) aims to provide the biomolecular researchers involved in taxonomic studies of environmental microbial communities with a simple and versatile workflow, comprehensive of all the fundamental bioinformatic steps, from raw sequence data handling to final taxonomic identification, to be used in HTS Meta-barcoding experiments. The BioMaS pipeline includes state-of-the-art available tools, such as FastQC [5], AmpliconNoise [6], BLAST [7], Bowtie2 [8], and TANGO [9,10], suitably tested and integrated with *ad hoc* designed Python scripts in order to manage HTS raw data, to convert them in suitable format for quality check and comparative analysis and, finally, to infer the taxonomical composition of the microbiome under investigation. All the mentioned software was selected among different available tools that were comparatively evaluated. In its current version, BioMaS allows the analysis of both bacterial and fungal composition and two alternative paths can be followed in order to process data obtained by Roche 454 GS FLX Titanium or Illumina MiSeq platforms, respectively.

BioMaS has been tested by comparing its performance with that of QIIME (Quantitative Insights Into Microbial Ecology) [11] and Mothur [12] in the analysis of an *in silico* simulated dataset of 16S rRNA bacterial gene V5-V6 and fungal ITS1 sequences provided with a curated taxonomical annotation.

## Methods and implementation

BioMaS carries out a sequential flow of analysis steps starting from the meta-barcode HTS raw data to provide a detailed characterization of the taxonomic complexity of the microbiome. All its analysis software have been implemented and integrated in an entirely automated workflow structured, in both 454 and Illumina data analysis versions, in consecutively running pre-built modules which basically accomplish the assessment of the sequencing data quality, their clustering according to the original samples, the reduction of sequence errors noise, the comparison with reference databases and, finally, the taxonomic binning and annotation.

### 454 data analysis pipeline

**Figure 1:**
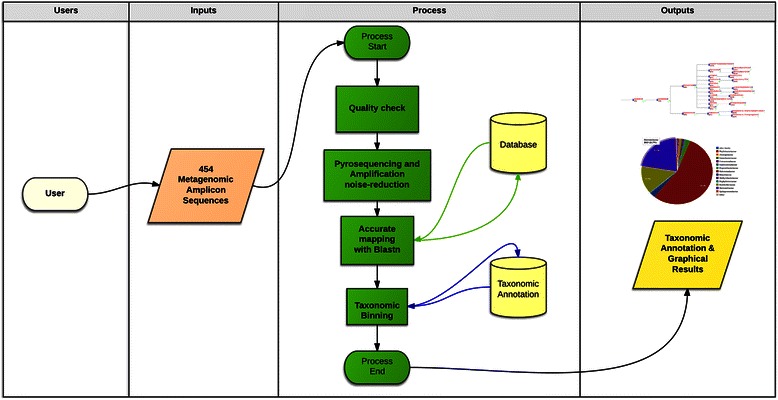
BioMaS 454 version. BioMaS workflow for the analysis of Roche 454 data.

The pipeline for Roche 454 data analysis includes four modules (Figure 1). The first one starts with the conversion of raw data (contained in the *sff* file, directly produced by the platform) to *fastq*, *fasta* and *sff.txt* formats, which are required by the subsequent analysis software included in BioMaS. The data in *fastq* format are then processed by FastQC, in order to obtain a statistical overview (e.g. distribution of the reads length, base composition, etc.) and quality snapshot of the sequence reads. The first module ends with the demultiplexing phase that is performed if multiple samples are sequenced in the same 454 run. Specifically, all the reads are screened and clustered according to the sample-specific indices (MID), appropriately included in the barcode amplicons during the preparation of the sequencing library [13].

In the second module the sequences are “cleaned” from the potential errors introduced during both PCR and pyro-sequencing (single nucleotide substitutions and overestimated homopolymers length) through a combination of software that include AmpliconNoise [6] and additional new scripts able to perform a final control of its outputs. The drastic reduction of sequence errors by means of AmpliconNoise represents a fundamental practice for considerably reducing the probability of biased taxonomic inferences.

In the third module the cleaned (denoised) sequences are aligned, through the BLASTN tool, against taxonomically annotated reference databases, specifically RDP II (Ribosomal Database Project II) [14] or GreenGenes [15], two collections of 16S rRNA sequences suitable for prokaryotic taxa identification or ITSoneDB [4], a collection of ITS1 sequences designed for supporting the taxonomic characterization of Fungi. The database similarity searching provides an *xml* output where, for each query sequence, the BLASTN hits are parsed using the following suitably pre-defined parameters: identity % (≥97%), query coverage % (≥70%), taxonomic information (matches to reference sequences with a complete taxonomic path are prioritized with respect to other matches, e.g. against an “uncultured bacterium”), and alignment bit-score (matches are recorded if their alignment bit score is not lower than 5% with respect to that of the best match). For each query sequence the list of significant hits is annotated in a “match file”. In the fourth module the match file is processed by TANGO [9,10], able to perform an optimal mapping of each of the sequences to the NCBI reference taxonomy [16] using a pre-computed guide tree representing the reference database. Finally, TANGO results are converted into a graphical tree and taxonomic rank-specific pie-charts representing the microbial complexity of the habitat under investigation by using the ETE environment [17].

### Illumina data analysis pipeline

**Figure 2:**
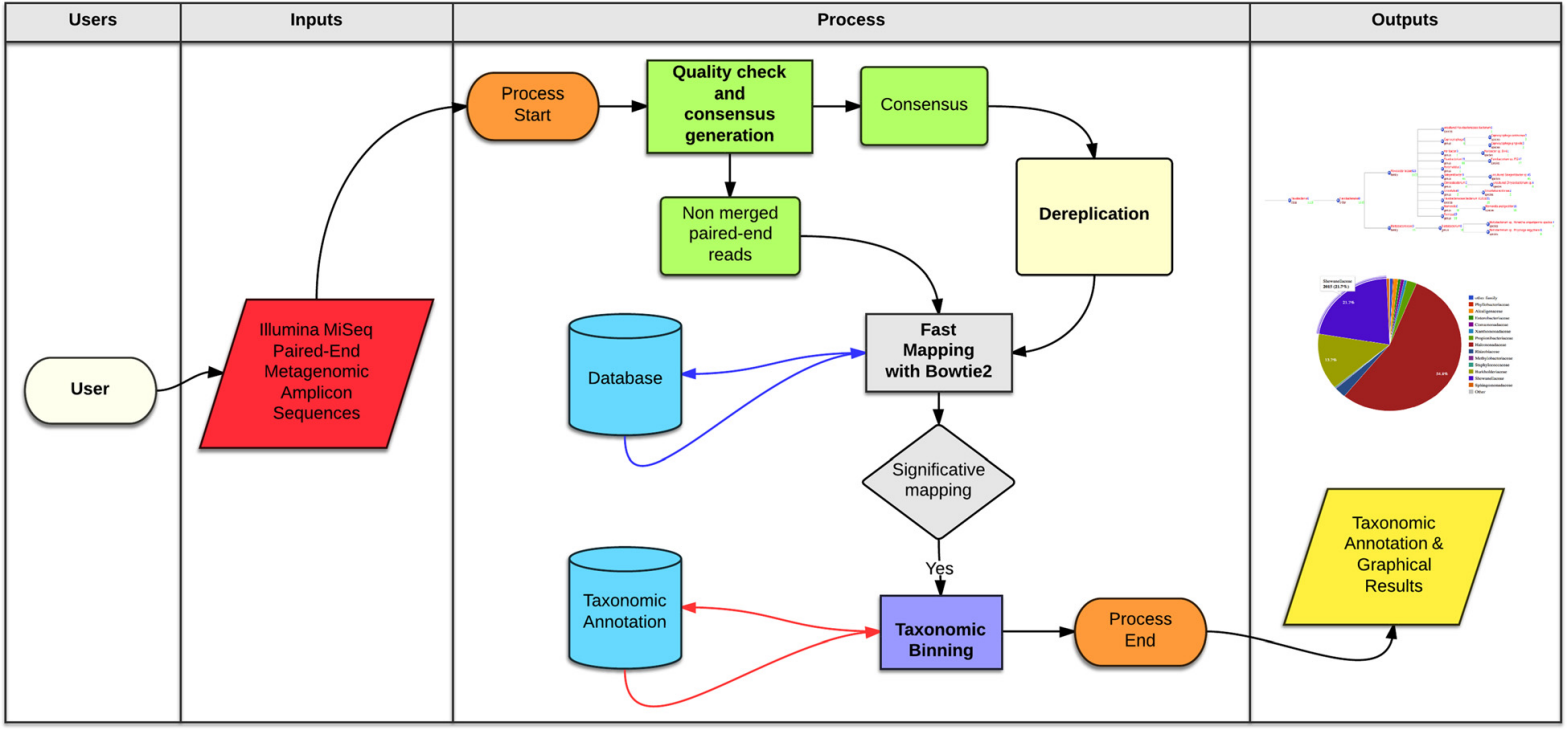
BioMaS Illumina version. BioMaS workflow for the analysis of Illumina paired-end reads.

The BioMaS workflow for Illumina data analysis preserves the same modular structure of the 454 version (Figure 2). The first module starts with the exploration of raw data that are directly produced in *fastq* format by the sequencing platform. In particular, FastQC performs the statistical and qualitative evaluation of the sequence data. Subsequently, the program Flash [18] merges overlapping paired-end reads to obtain a single consensus sequence if the length of the overlapping region and the number of observed mismatches fit specific default thresholds eventually customizable by the user. The obtained consensus sequences are then dereplicated by using Usearch [19]. The non-overlapping reads pairs are analysed separately, by means of *Trim Galore!* [20], in order to remove low quality regions (Phred score ≤ 25). Also, pairs containing reads shorter than 50 nt are removed. Merged and paired-end sequences are both mapped to the taxonomically annotated databases implemented in BioMaS, namely RDP II [14], Greengenes [15] and ITSoneDB [4], by means of Bowtie2 [8], which was selected considering its ability to map both single and paired-end sequences, its mapping accuracy, and execution speed. The resulting alignment data are stored in a *sam* file. Then, for each query sequence (or paired-end sequences), the mapping hits are parsed according to identity %, query coverage % and taxonomic information and the most significant ones are annotated in a “match file”. In the final module the match file is processed by TANGO [9,10] in order to map the sequences to the NCBI taxonomy. Finally, the resulting microbiome composition is graphically rendered by means of a graphical tree and taxonomic rank-specific pie-charts by using the ETE environment [17].

BioMaS results include also a textual *csv* file, summarizing the taxonomic assignments, that the user can exploit to merge the taxonomic classification data from different samples by means of the BioMaS Post-Processing Tools, in order to produce a file suitable for the comparative analysis through METAGENassist [21].

### Job Submission Tool (JST)

Bioinformatics applications for the analysis of environmental microbial communities are expensive in terms of computational resources. For this reason, the Grid/Cloud technology appears to fit the requirements of such applications. For example, those technologies are able to provide easily and seamlessly the needed computational power as well as the storage resources to record the produced data. JST [22] is a job management tool particularly useful to manage the submission and monitoring of applications, when a large number of independent executions are needed to solve a given problem. This tool is able to work on different infrastructures like:

- the EGI Grid infrastructure,
- dedicated servers,
- local batch farms,
- IaaS (Infrastructure as a Service) based cloud resources.

By means the JST capabilities it is also possible to exploit all the supported computing resources within a workflow manager like Taverna, LONI and Galaxy.

BioMaS service was implemented with two different web interfaces, both of them are exploiting the JST web service interface:

- Liferay Portal
- Galaxy Workflow Manager

The JST architecture is described in Additional file 1: Figure S1.

#### Liferay portal

A web interface for BioMaS has been realized based on the Catania Science Gateway (http://www.catania-science-gateways.it). This portal is based mainly on Liferay framework that allows the developers to build a simple portlet component that interacts with the JST on the back-end. In this way, the complexity of dealing with the different computational platforms is hidden by JST and the developers can concentrate the effort on providing simple and powerful graphical interfaces. On the INFN resources, provided by the ReCaS project (http://www.pon-recas.it/), a Science Gateway based on Liferay has been installed, where a brand new portlet has been developed to support BioMaS. Using this Java portal the user is able to submit a new BioMaS analysis and easily check the status of the requested computation. Furthermore, the user receives a mail notification where the JST backend component reports the final status of the job and the link where the user can download the output results of the BioMaS execution. This portal also supports several kind of authentications, including those related to social networks accounts (Facebook, Google, Twitter, etc.) or the authentication mechanism already used at company and institutes (University, research institutions, etc.).

#### Galaxy workflow manager

The Illumina version of BioMaS has been also implemented in Galaxy and exposed as service in the Biodiversity Virtual eLaboratory project (BioVeL, http://www.biovel.eu/). Galaxy [23-25] is an open platform written in Python implementing a Workflow Management System (WFMS) designed to fulfil the requirements of the Bioinformatics community for data-intensive computational analysis and data integration, allowing to build complex workflows and to document, share and publish results. The Galaxy system, increasingly used by researchers, is a web-based application not requiring the installation of local client software by the user. On the INFN computational resources, a custom version of Galaxy is installed in order to support BioMaS.

In particular, in this Galaxy instance, an ad-hoc workflow has been developed and customized.

This workflow is characterized by three macro-modules developed by INFN:

1. upload input files
2. submit analysis
3. recover results

Using these simple building blocks also the end-users should be able to build new workflows exploiting the basic application provided as SaaS (Software as a Service) by mean of JST interface, and composing them to build more complex and high-level analyses. A generic instance of JST is used in order to exploit all the available resources needed to schedule and execute the different steps needed by BioMaS tool. Both the JST itself and the computational resources are provided exploiting modern IaaS Cloud Computing technology, in order to be able to guarantee the scalability and the reliability needed by a service that is publicly exposed to the end-users. In the INFN Galaxy instance the Galaxy BioMaS workflow package has been created and shared with the BioMaS users. These users can import and run the workflow in their private Galaxy environment through the web interface. On this instance is already available a BioMaS workflow suitably created to perform the analysis described in this paper. In Additional file 2: Figure S2 an example is shown of a BioMaS workflow submission in the Galaxy instance.

## Results and discussion

### BioMaS web service

BioMaS has been implemented as web-application at https://recasgateway.ba.infn.it/ and its Illumina version is also available in Galaxy at http://galaxy.cloud.ba.infn.it:8080. After a registration step, which is required to use both systems, the user can login and use the pipeline by simply uploading the *fastq* or *sff* files for Illumina and 454, respectively, containing the reads to be analysed, and providing a job name and a valid e-mail address. The results of the analysis will be sent to the user e-mail, and will consist of a tree representation of the microbial composition and interactive taxonomic level-specific pie-charts. Moreover, a tabular file will be supplied in order to allow comparative analysis between different samples by using the BioMaS Post-Processing Tools and METAGENassist [21]. The main idea behind the construction of the BioMaS web service was to obtain a fully automated analysis system in which the user just needs to upload the data produced by the sequencing platform to get a simple picture of the taxonomic composition of the original sample. The system is therefore readily accessible also to researchers with limited bioinformatics skills as they do not have to worry about using, integrating, and, usually, designing the software necessary to perform the intermediate stages of the workflow. Notwithstanding the intention to preserve the simple use of the web service, the introduction of some points of parameterization by the user are planned in the near future. In particular, other curated reference rRNA 16S databases, such as, for example, SILVA [26], will be supported in order to enable users to select their favourite one.

### BioMaS performance

The taxonomic assignment performance of BioMaS was compared to that of two popular tools, QIIME [11] and MOTHUR [12], both developed for the analysis and comparison of microbial communities primarily based on high-throughput sequencing of their meta-barcode amplicons. The assignment benchmark was performed for both Bacteria, by considering the V5-V6 region of 16S rRNA gene as taxonomic marker, and for Fungi, by adopting the ITS1 region. The releases 1.8.0 of QIIME and 1.34.4 of Mothur were used in the tests. The same release (13.8) of GreenGenes was used as the reference database for all the three pipelines in the Bacteria case study. In the case of Fungi we were unable to use the same reference dataset, and ITSoneDB [4] database (updated to release 202 of GenBank) was used for BioMaS, and UNITE [27] database was used instead for both QIIME (release 12_11) and Mothur (release 2014-12-30).

Virtual collections of meta-barcode 454 and Illumina sequences were generated for Bacteria and Fungi. We detail in the following the building procedure of the virtual collections. Initially, all the possible V5-V6 and ITS1 sequences were extracted from RefSeq database [28,29], by performing an “in silico PCR” by means of the PatSearch tool [30,31] and the standard universal primers commonly adopted for the amplification of these regions (forward: TTAGATACCCYGGTAGTCC, reverse: ACGAGCTGACGACARCCATG [32] for V5-V6 and Forward: GAACCWGCGGARGGATCA, reverse: GCTGCGTTCTTCATCGATGC [33] for ITS1). Subsequently, amplicon sequences belonging to 102 bacterial species and 101 fungal species were randomly extracted, but forcing the inclusion of co-generic species (58 and 60 for bacteria and fungi, respectively). The length distribution of extracted sequence amplicons, as expected [32], is narrower for V5-V6 (around 300 bp) than for ITS1 sequences, which show a remarkable length variability (Additional file 3: Figure S3).

The simulated dataset of Illumina MiSeq 250×2 paired-end reads was obtained by applying ART [34], a tool able to generate synthetic HTS reads, for both the V5-V6 and ITS1 regions. This procedure allowed us to obtain 510,000 V5-V6 paired-end reads and 505,000 ITS1 paired-end reads. The simulated dataset of 454 reads was obtained by applying ART [34] and Flowsim [35], able to simulate flowgram data starting from FASTA sequences, for both the V5-V6 and ITS1 regions. This procedure allowed us to obtain 24,134 V5-V6 sequences and 23,819 ITS1 sequences.

**Figure 3:**
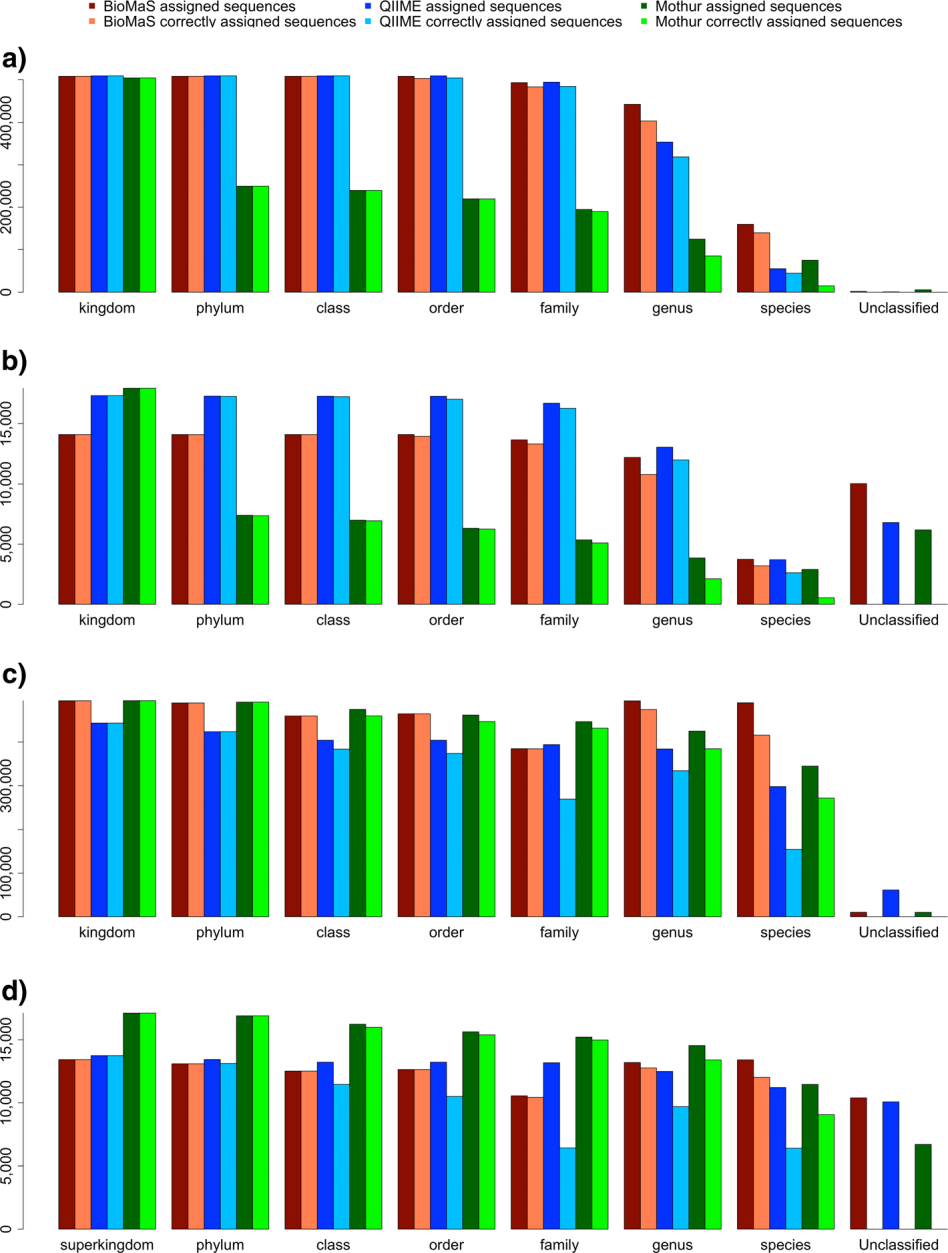
Results of the comparative analysis of BioMaS, QIIME and Mothur platforms. Red, blue and green columns indicate the total number of assigned sequences by BioMaS, QIIME and Mothur, respectively. Alternating with the first ones, light red, light blue and light green columns indicate the number of sequences that are correctly annotated by the three methods. The figure is divided in 4 sections, as follows: **a)** Illumina Bacteria test case, **b)** Roche 454 Bacteria test case, **c)** Illumina Fungi test case, and **d)** Roche 454 Fungi test case.

A taxonomical coverage overview of these virtual collections is provided in Additional file 4: Table S1. The results of the comparative assessment of BioMaS, QIIME and Mothur are shown in Figure 3, where the number of total assigned and of correctly assigned reads (according to the known taxonomic label of sequences belonging to the simulated dataset) are plotted.

As regards the Bacteria benchmark with Illumina simulated sequences dataset, QIIME classified 494,195 sequences (97.98% correctly assigned) at family level, 353,553 sequences (90.13% correctly assigned) at genus level, and 54,707 sequences (82.10% correctly assigned) at the species level. Mothur classified 194,563 sequences (97.44% correctly assigned) at family level, 124,547 sequences (68.07% correctly assigned) at genus level and 74,660 sequences (20.07% correctly assigned) at the species level of the same starting dataset. BioMaS outperformed both QIIME and Mothur at lower taxonomic levels, in particular at the genus and species level, as it was able to classify 493,089 sequences (97.97% correctly assigned) at family level, 442,735 sequences (91.12% correctly assigned) at genus level and 159,554 sequences (87.49% correctly assigned) at species level (see also Additional file 5: Table S2). This trend was confirmed by the results of the benchmark on Fungi (simulated Illumina dataset) with BioMaS outperforming QIIME and Mothur at family, genus, and species levels (see also Additional file 5: Table S2).

As regards the 454 simulated sequences dataset in the Bacteria benchmark (see Additional file 5: Table S2) all three pipelines seem to have worse performances in terms of number of classified sequences compared to the size of the original simulated dataset. However, in this case BioMaS still remains the best method at the species level in terms of total and correctly classified sequences, but QIIME performs better at higher taxonomic levels. Conversely, when 454 sequences are assigned to fungal taxa, a similar trend is observed with BioMaS outperforming the other two pipelines at lower taxonomic ranks, but in this case Mothur perform better at higher taxonomic levels (see Additional file 5: Table S2).

The number of not classified sequences, calculated as difference between the number of sequences included in the simulated dataset and the number of those assigned to any rank (Additional file 5: Table S2) may account for some of the performance differences described before. The variable amount of unclassified sequences in the simulated benchmark depends on the effectiveness of the assignment strategies, specific for each pipeline. Notably, in real life applications, unclassified sequences may likely derive from the lack of sufficiently similar sequences in the reference databases.

Moreover, in order to compare the ability of BioMaS, QIIME, and Mothur to reliably assess the quantitative occurrence of each taxon in the simulated dataset (Additional file 4: Table S1), True Positive Rate (TPR), True Negative Rate (TNR), False Positive Rate (FPR) and False Negative Rate (FNR) were measured for each of the three pipelines under investigation.

In order to perform this analysis, each simulated read was associated to the corresponding taxonomic path in the Greengenes taxonomy for the bacterial species and in the NCBI taxonomy for the fungal species. Then, the expected taxonomy of each read has been compared to that obtained by the three different methods. This allowed us to calculate for each taxon *i,* the following parameters:

- *TP*_*i*_ is the number of reads belonging to *i* and assigned to *i*;
- *FN*_*i*_ is the number of reads belonging to *i* but not assigned to *i*;
- *FP*_*i*_ is the number of reads belonging to *j* (*j* ≠ *i*) but assigned to *i*;
- *TN*_*i*_ is the number of reads belonging to *j* (*j* ≠ *i*) and assigned to *j*.

TPR, TNR, FPR and FNR were then calculated for each taxon *i* as follows:

- TPR (Sensitivity) corresponds to the proportion of reads belonging to *i* and correctly assigned to the node i:

$$ TP{R}_i=\frac{T{P}_i}{\left(T{P}_i+F{N}_i\right)} $$

- TNR (Specificity) measures the proportion of reads that are correctly not assigned to the node *i* compared to all the ones that are expected not to belong to *i* (*TN*_*i*_ + *FP*_*i*_):

$$ TN{R}_i=\frac{T{N}_i}{\left(T{N}_i+F{P}_i\right)} $$

- FPR measures the proportion of reads that are incorrectly assigned to the node *i* compared to all the ones that are expected not to belong to *i* (*TN*_*i*_ + *FP*_*i*_):

$$ FP{R}_i=\frac{F{P}_i}{\left(T{N}_i+F{P}_i\right)} $$

- FNR is the proportion of reads belonging to the node *i* but not assigned to *i*:

$$ FN{R}_i=\frac{F{N}_i}{\left(T{P}_i+F{N}_i\right)} $$

Finally, for each rank (from kingdom to species) the average assignment performance was calculated as follows:

$$ TPR=\raisebox{1ex}{${\displaystyle \sum }TP{R}_i$}\!\left/ \!\raisebox{-1ex}{${\displaystyle \sum }i$}\right. $$

$$ TNR=\raisebox{1ex}{${\displaystyle \sum }TN{R}_i$}\!\left/ \!\raisebox{-1ex}{${\displaystyle \sum }i$}\right. $$

$$ FPR=\raisebox{1ex}{${\displaystyle \sum }FP{R}_i$}\!\left/ \!\raisebox{-1ex}{${\displaystyle \sum }i$}\right. $$

$$ FNR=\raisebox{1ex}{${\displaystyle \sum }FN{R}_i$}\!\left/ \!\raisebox{-1ex}{${\displaystyle \sum }i$}\right. $$

The results of the above described evaluations, performed for BioMaS, QIIME and Mothur, are shown in Table 1 and Additional file 6: Table S3.

**Table 1:** **Statistics of the quantitative evaluation of the BioMaS, QIIME and Mothur pipelines**

**a) Illumina bacteria test case**												
	**BioMaS**				**QIIME**				**Mothur**			
**Rank**	**TPR**	**TNR**	**FPR**	**FNR**	**TPR**	**TNR**	**FPR**	**FNR**	**TPR**	**TNR**	**FPR**	**FNR**
Kingdom	100.00	0.00	0.00	0.00	100.00	0.00	0.00	0.00	100.00	0.00	0.00	0.00
Phylum	100.00	100.00	0.00	0.00	100.00	0.00	0.00	0.00	44.47	100.00	0.00	55.53
Class	100.00	100.00	0.00	0.00	100.00	100.00	0.00	0.00	44.35	100.00	0.00	55.65
Order	97.62	99.98	0.02	2.38	97.62	99.98	0.02	2.38	39.13	100.00	0.00	60.87
Family	94.23	99.96	0.04	5.77	94.23	99.96	0.04	5.77	36.50	99.98	0.02	63.50
Genus	87.00	99.90	0.10	13.00	66.03	99.91	0.09	33.97	16.91	99.89	0.11	83.09
Species	87.28	99.92	0.08	12.72	28.13	99.95	0.05	71.87	9.38	99.73	0.27	90.63
**b) Roche 454 bacteria test case**												
	**BioMaS**				**QIIME**				**Mothur**			
**Rank**	**TPR**	**TNR**	**FPR**	**FNR**	**TPR**	**TNR**	**FPR**	**FNR**	**TPR**	**TNR**	**FPR**	**FNR**
Kingdom	100.00	0.00	0.00	0.00	100.00	0.00	0.00	0.00	100.00	0.00	0.00	0.00
Phylum	99.99	100.00	0.00	0.01	99.23	100.00	0.00	0.77	35.63	99.99	0.01	64.37
Class	99.99	100.00	0.00	0.01	96.26	100.00	0.00	0.89	34.20	99.99	0.01	65.80
Order	97.61	99.99	0.01	2.39	94.16	99.98	0.02	3.46	29.98	99.99	0.01	70.02
Family	91.80	99.98	0.02	6.27	87.49	99.97	0.03	8.66	26.61	99.98	0.02	71.46
Genus	82.14	99.94	0.06	16.32	68.53	99.94	0.06	28.40	11.94	99.91	0.09	86.52
Species	68.88	99.96	0.04	24.87	42.58	99.88	0.12	51.17	8.81	99.80	0.20	84.94
**c) Illumina fungi test case**												
	**BioMaS**				**QIIME**				**Mothur**			
**Rank**	**TPR**	**TNR**	**FPR**	**FNR**	**TPR**	**TNR**	**FPR**	**FNR**	**TPR**	**TNR**	**FPR**	**FNR**
Kingdom	100.00	0.00	0.00	0.00	100.00	0.00	0.00	0.00	100.00	0.00	0.00	0.00
Phylum	100.00	100.00	0.00	0.00	97.15	99.51	0.49	2.85	99.51	100.00	0.00	0.49
Class	99.73	100.00	0.00	0.27	72.77	99.36	0.64	27.23	92.09	99.76	0.24	7.91
Order	96.30	100.00	0.00	3.70	65.15	99.66	0.34	24.85	85.92	99.90	0.10	14.08
Family	93.20	100.00	0.00	6.80	66.36	99.23	0.77	25.64	84.28	99.94	0.06	15.72
Genus	94.47	99.92	0.08	5.53	65.50	99.67	0.33	24.98	75.07	99.84	0.16	23.34
Species	83.55	99.90	0.10	16.45	30.69	99.35	0.65	57.43	53.74	99.79	0.21	44.28
**d) Roche 454 fungi test case**												
	**BioMaS**				**QIIME**				**Mothur**			
**Rank**	**TPR**	**TNR**	**FPR**	**FNR**	**TPR**	**TNR**	**FPR**	**FNR**	**TPR**	**TNR**	**FPR**	**FNR**
Kingdom	100.00	0.00	0.00	0.00	99.10	0.00	0.00	0.00	100.00	0.00	0.00	0.00
Phylum	98.64	100.00	0.00	1.36	97.96	99.89	0.11	2.04	99.10	100.00	0.00	0.90
Class	99.44	100.00	0.00	0.56	72.12	99.76	0.24	27.88	93.72	99.92	0.08	6.28
Order	96.35	100.00	0.00	3.65	56.27	99.82	0.18	20.40	83.79	99.97	0.03	16.21
Family	92.79	100.00	0.00	7.21	56.00	99.62	0.38	22.00	84.43	99.98	0.02	15.57
Genus	94.44	99.97	0.03	5.56	57.18	99.86	0.14	17.42	75.60	99.93	0.07	24.40
Species	89.25	99.96	0.04	10.75	33.75	99.84	0.16	41.50	53.24	99.92	0.08	46.76

Average True Positive Rate (TPR), average True Negative Rate (TNR), average False Positive Rate (FPR) and average False Negative Rate (FNR) values for all the considered ranks (from kingdom to species) are shown for BioMaS, QIIME and Mothur computation. The table is divided in 4 sections, as follows: a) Illumina Bacteria test case, b) Roche 454 Bacteria test case, c) Illumina Fungi test case, and d) Roche 454 Fungi test case.

With regard to the highest taxonomic ranks (kingdom, phylum and class), BioMaS and QIIME perform better than Mothur in the Bacteria benchmark with both Illumina and 454 datasets. Conversely, for the deeper ranks, particularly at the species level, BioMaS outperforms both QIIME and MOTHUR in all the meta-barcode/NGS platform dataset arrangements, particularly in sensitivity, quantified as TPR.

BioMaS showed a higher performance in terms of sensitivity at each taxonomic rank in the Fungi benchmark with both NGS platforms datasets, compared to QIIME and Mothur.

In both Bacteria and Fungi cases, the FNR increase from higher to lower ranks due to the greater difficulty, for all the systems, to discriminate sequences belonging to very closely related taxa. Nevertheless, also in this case BioMaS performed better, particularly at lower taxonomic ranks, in all the meta-barcode/NGS platform dataset arrangements. Finally, the performance of BioMaS, QIIME and MOTHUR is comparable as regards the TNR (Specificity) and the FPR rates evaluation.

Finally, in order to verify how much the assignment performance of BioMaS is biased by taxonomical composition of reference collections, an additional test has been performed by randomly removing from them (i.e. Greengenes and ITSoneDB) 50% of the genera included in the simulated collections. The obtained results were analysed to verify if the sequences belonging to species not represented in the reference database were not assigned at all or assigned at higher taxonomic ranks, such as the family level. In the bacterial benchmark, 22,221 reads, (6.44% of the 345,000 Illumina reads belonging to genera that were removed from Greengenes database) were erroneously assigned, at any taxonomic level. The remaining reads were not classified at all (231,131 reads, 66,99%) or classified at least at family level (91,658, 26,57%). In the Fungi benchmark, 70,190 Illumina reads (19.23% of the 365,000 Illumina reads belonging to genera that were removed from ITSoneDB), were wrongly assigned. The remaining sequences were not classified at all (294,810, 80,77%). A further analysis of the fungal data showed that 38,739 of the unassigned sequences were classified as “uncultured fungus”. This was mainly due to incomplete taxonomic information in the reference database. In conclusion, for both Bacteria and Fungi, the proportion of unclassified sequences is mostly dependent on the taxonomic coverage of the reference database, whereas the proportion of BioMaS wrong assignments is quite low (see Table 1 and Figure 3).

The computational time needed for BioMaS execution mainly depends on the size of the analysed dataset and the number CPU used in the GRID environment. For example, for the specific V5-V6 datasets used in the benchmark, in which 20 processors have been engaged, the execution times were 1 hour for Illumina and 2 days for 454 data, on average. In particular, the greater time required for 454 depended essentially on computational effort dedicated to AmpliconNoise execution. The Illumina version of BioMaS has been applied in a recently published study [36], to a real dataset aimed at the taxonomic characterization of bacterial communities inhabiting a marine coastal lagoon (Varano, Adriatic Sea). In this case the mean time to process a real 16S rRNAV5-V6 dataset of about 1,300,000 sequences was about 24 hours. We tested also the scalability of the code in the Grid and Cloud environment, verifying that it is quite linear: this means that the more CPUs will be used the faster the execution of the application will be.

## Conclusions

Nowadays the metagenomic surveys based on the increasingly advanced HTS technologies gives rise to ambitious challenges for the bioinformatic analysis of the data. Researchers face with the non-trivial difficulty to select, use and integrate all the most suitable tools to obtain correct inferences, and in some cases new software development is needed for such purpose. BioMaS includes a wide range of bioinformatics tools, carefully selected and tested, integrated into an automated workflow, allowing the user to quickly obtain a comprehensive view of the deep taxonomic complexity of the environmental samples under investigation, effectively represented by means of simple graphical outputs, starting directly from meta-barcode HTS raw datasets. Thanks to its global way to deal with this issue, BioMaS allows user-friendly analyses not requiring specific computer skills and, at the same time, providing easily interpretable results.

The benchmark results demonstrated that BioMaS is a valid tool for the deep taxonomic assignment of metagenomic amplicons HTS datasets. Indeed, it outperformed both QIIME and Mothur mainly at lower taxonomic levels. Moreover, the greater sensitivity observed for BioMaS at all the taxonomic levels for Fungi and mainly at deeper taxonomic ranks for Bacteria, compared to QIIME and Mothur, highlights its great accuracy in revealing also the quantitative differences between the various species represented in the starting sequences dataset. This property is very important in the metagenomic studies in which microbial population dynamics are deeply monitored in relation to a number of variables, such as environmental, temporal or host physio-pathologic ones.

Finally, the comprehensive microbial taxonomy coverage of the simulated sequences used in the benchmark comparison and their widespread correct assignment made by BioMaS, suggests that this pipeline has a significant universal potential, allowing the effective analysis of very diverse microbial environments.

### Availability and requirements

BioMaS is available upon registration as web-application at https://recasgateway.ba.infn.it/ and in the Galaxy framework at http://galaxy.cloud.ba.infn.it:8080.

## Additional files

Additional file 1: Figure S1. JST architecture scheme.
Additional file 2: Figure S2. Example of BioMaS workflow submission in Galaxy.
Additional file 3: Figure S3. Length distribution of in silico V5-V6 and ITS1 amplicons obtained by applying PatSearch to the sequences extracted from RefSeq database.
Additional file 4: Table S1. Taxonomical representation of the species used to build the virtual dataset. The reference taxonomy of eight fungal species (e.g. *Trinosporium guianense*) lacks the information of the intermediate ranks (e.g. family).
Additional file 5: Table S2. Results of the comparative evaluation of the BioMaS, QIIME and Mothur performances. In Fungi test case the reads assigned at family level are underestimated for eight species because of the lack of this rank in the used reference taxonomy.
Additional file 6: Table S3. Extended statistics of the quantitative evaluation of the BioMaS, QIIME and Mothur pipelines reported in Table 1, where the relevant values for 25^th^, 50^th^ and 75^th^ percentiles are also reported.

## Abbreviations

HTS: High-Throughput Sequencing
PCR: Polymerase chain reaction
ITS: Internal transcribed spacer
BioMaS: Bioinformatic analysis of Metagenomic ampliconS
TANGO: Taxonomic assignment in metagenomics
QIIME: Quantitative insights into microbial ecology
rRNA: Ribosomal ribonucleic acid
SFF: Standard flowgram format
MID: Multiplex identifier
RDP: Ribosomal Database Project
XML: EXtensibile Markup Language
NCBI: National Center for Biotechnology Information
ETE: Environment for tree exploration
JST: Job Submission Tool
EGI: European GRID Infrastructure
IaaS: Infrastructure as a Service
SOAP: Simple Object Access Protocol
REST: Representational State Transfer
WFMS: Workflow Management System
PON: Programmi Operativi Nazionali
TPR: True Positive Rate
TNR: True Negative Rate
FPR: False Positive Rate
FNR: False Negative Rate
FTP: File Transfer Protocol
