# Supplementary figures and images for: BioMaS: a modular pipeline for Bioinformatic analysis of Metagenomic AmpliconS
Source: BMC Bioinformatics. 2015 Jul 1;16:203. doi: 10.1186/s12859-015-0595-z (PMC4486701; doi:10.1186/s12859-015-0595-z)

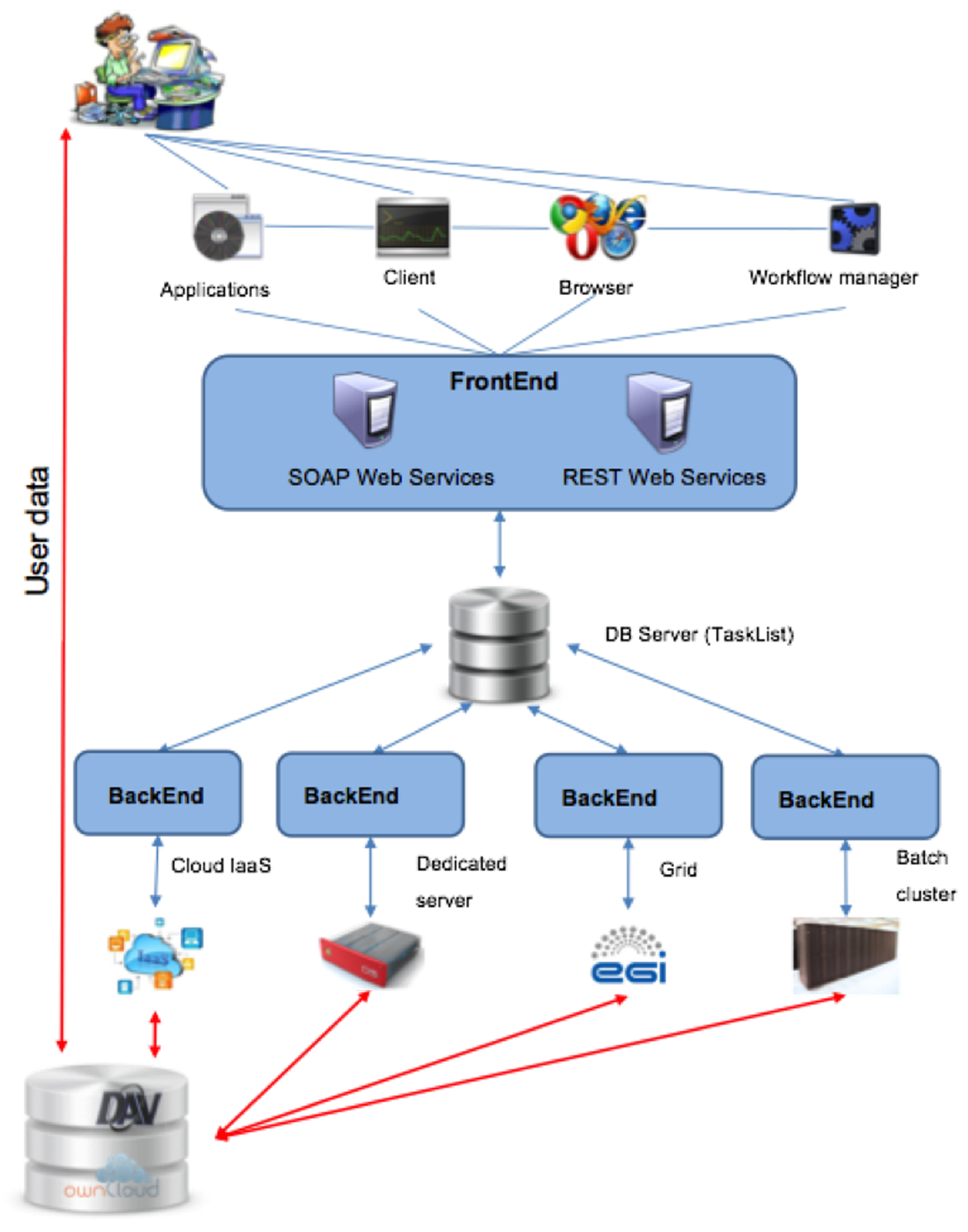

Supplement: Additional file 1: Figure S1. — JST architecture scheme. [file 12859_2015_595_MOESM1_ESM.png]

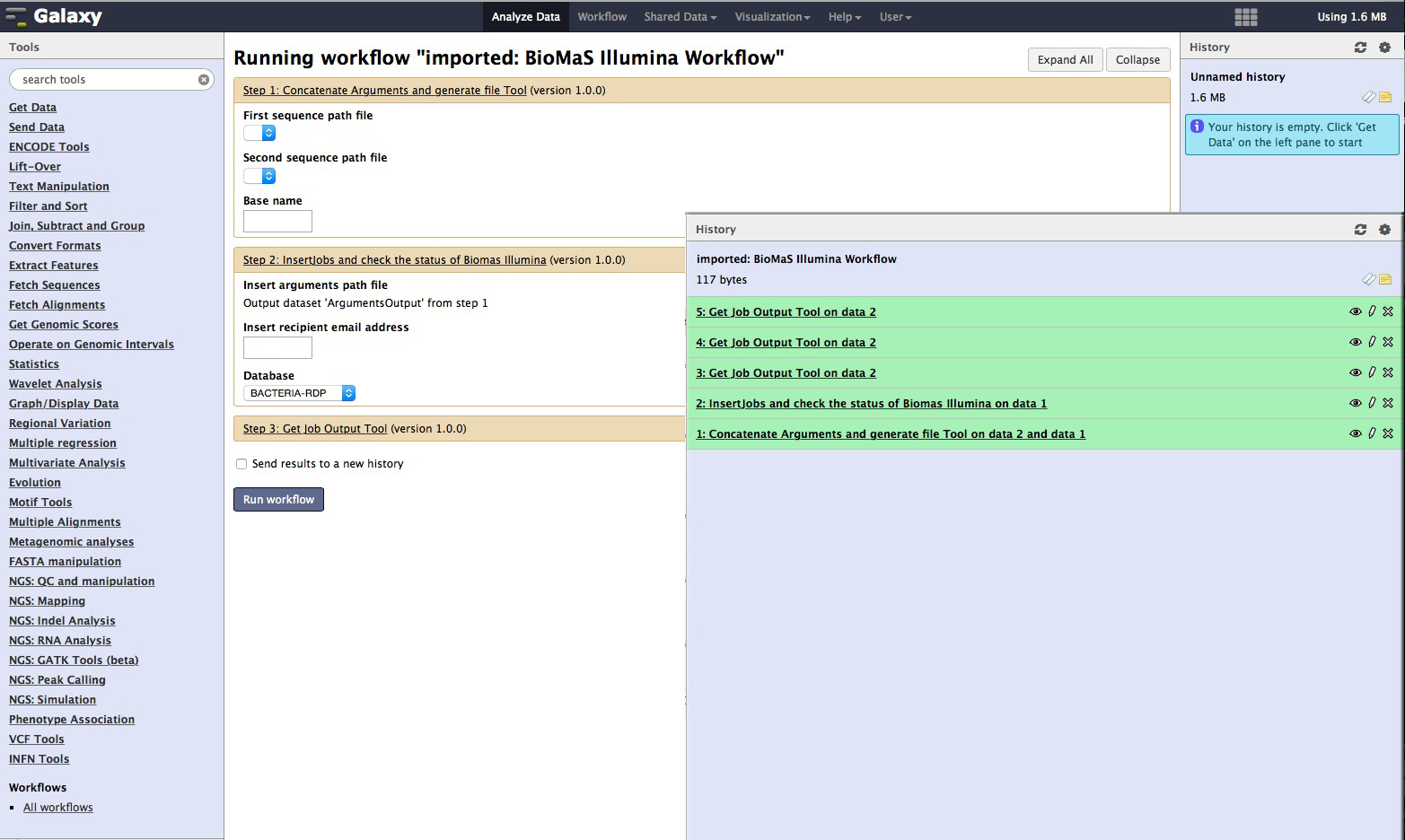

Supplement: Additional file 2: Figure S2. — Example of BioMaS workflow submission in Galaxy. [file 12859_2015_595_MOESM2_ESM.png]

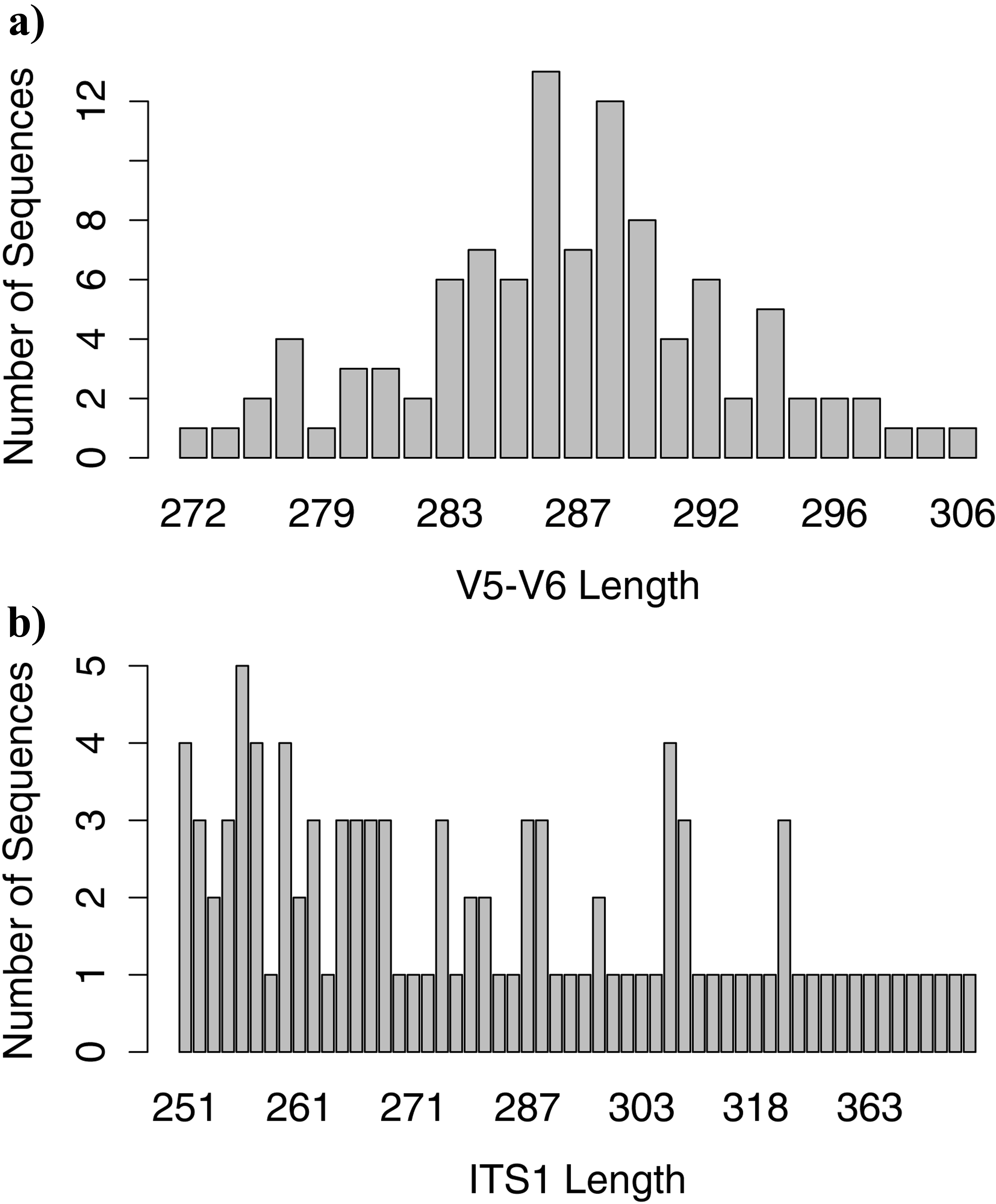

Supplement: Additional file 3: Figure S3. — Length distribution of in silico V5-V6 and ITS1 amplicons obtained by applying PatSearch to the sequences extracted from RefSeq database. [file 12859_2015_595_MOESM3_ESM.png]
